# Isolation and Characterization of Primary DMD Pig Muscle Cells as an *In Vitro* Model for Preclinical Research on Duchenne Muscular Dystrophy

**DOI:** 10.3390/life12101668

**Published:** 2022-10-21

**Authors:** Tina Donandt, Stefan Hintze, Sabine Krause, Eckhard Wolf, Benedikt Schoser, Maggie C. Walter, Peter Meinke

**Affiliations:** 1 Friedrich-Baur-Institute at the Department of Neurology, University Hospital, Ludwig-Maximilians-Universität München, 81377 Munich, Germany; 2 Chair for Molecular Animal Breeding and Biotechnology, Gene Center and Department of Veterinary Sciences, Ludwig-Maximilians-Universität München, 81377 Munich, Germany

**Keywords:** Duchenne muscular dystrophy, muscle cell culture, DMD pig, DMD, dystrophin, porcine muscle cells

## Abstract

Duchenne muscular dystrophy (DMD) is the most frequent genetic myopathy in childhood and leads to progressive muscle atrophy, weakness, and premature death. So far, there is no curative treatment available. Therapeutic development from bench to bedside takes time, and promising therapies need to be tested in suitable preclinical animal models prior to clinical trials in DMD patients. Existing mouse and dog models are limited with regard to the comparability of the clinical phenotype and the underlying mutation. Therefore, our group established a tailored large animal model of DMD, the DMD pig, mirroring the human size, anatomy, and physiology. For testing novel approaches, we developed a corresponding *in vitro* model, facilitating preclinical testing for toxicity, dosing, and efficacy, which we describe here. We first extracted primary muscle cells from wild-type and DMD pigs of different age groups and characterized those cells, then improved their differentiation process for identification of dystrophin and utrophin in myotubes. Our porcine *in vitro* model represents an important step for the development of novel therapeutic approaches, which should be validated further to minimize the need for living animals for bioassays, and thereby support the ‘3R’ (replace, reduce, refine) principle, as fewer animals have to be raised and treated for preclinical trials.

## 1. Introduction

Duchenne muscular dystrophy (DMD) is the most frequent genetic myopathy in childhood and leads to progressive muscle atrophy, weakness, and premature death. DMD is caused by mutations in the *DMD* gene encoding dystrophin, a cytoplasmic protein that enables the strength, stability, and functionality of myofibers. Disease-causing mutations, most frequently deletions, typically result in loss of dystrophin [1]. Patients frequently lose ambulation between age 9 and 12. With the loss of ambulation, cardiac and respiratory involvement occurs. Life expectancy is reduced to 30–40 years, although multidisciplinary symptomatic and surgical treatment has considerably improved survival within the last two decades, mainly by standard of care treatment with corticosteroids and non-invasive ventilation [2].

Until today, there is no cure, although DMD is within the target focus of new gene-based therapies [3]. There are two predominant strategies for future treatment, dystrophin-restoring and dystrophin-independent treatment approaches. So far, there are numerous dystrophin-restoring strategies under investigation [4]: exon skipping via antisense oligonucleotides (AONs) in patients with specific out-of-frame deletions that can be spliced to a shorter but in-frame mRNA [5]; stop-codon read-through targeting nonsense mutations [6]; gene replacement therapies via AAV vectors [7], and gene editing approaches via CRISPR/Cas9 systems [8,9]; or replacement of dystrophin with a surrogate protein, e.g., utrophin [10]. New generation steroids such as vamorolone, histone deacetylase- and NF kappa B-inhibitors, myosin modulators, and agents along the myostatin inhibiting pathway are promising future agents independent of dystrophin and the underlying mutation [11].

Existing mouse and dog models have been crucial to understand the pathophysiologic background of DMD and to develop treatment strategies, but are limited in mirroring the clinical phenotype and the type of mutation [12]. The DMD pig lacking *DMD* exon 52, which has been developed by our group, displays a progressive and markedly accelerated muscular dystrophy, and a similar phenotype compared to the human disease with proximal muscle weakness, cardiac and respiratory involvement [11].

The development of molecular therapies usually requires the usage of *in vitro* models before they are being tested in animal models. This allows an early assessment and, if necessary, modification of the respective approach, thus reducing the number of animals used. Therefore, the choice of the *in vitro* model to be used is an important one. While muscle cells are the obvious choice for DMD, it becomes more complicated choosing the species the muscle cells are gained from. Muscle cell cultures gained directly from DMD patients appear to be the preferred choice, but there is very limited access to these as diagnosis for DMD today does no longer require a muscle biopsy but is based on genetic diagnosis [13]. Thus, methods like immortalization of human muscle cells [14], CRISPR/Cas9 gene editing to create DMD models, using immortalized cells [15], or reprogramming of different cell types (e.g., urine derived cells, [16]) have been employed [17]. However, benefits of these model systems should be weighed against possible disadvantages, since cellular senescence is a contributing factor in DMD [18], a readout that will be lost in immortalized cells. Another consideration is the animal model used later on—it is preferable testing the respective therapy in a model system derived from the animal model of choice due to possible species-specific characteristics. Here, we describe our experience in extracting, cultivating, and differentiating primary muscle cell cultures from DMD and wild-type pigs as an *in vitro* model for DMD.

## 2. Materials and Methods

### 2.1. Animals

The generation and characterization of the DMD pig have been published elsewhere [12,19]. In short, a German Landrace and Swabian-Hall mix sow with a heterozygous *DMD* exon 52 deletion (*DMD*Δ52) was interbred with wild-type boars, resulting in the following offspring: male *DMD*Δ52 pigs (DMD^Y/−^ or DMD pig), heterozygous *DMD*Δ52 carrier pigs (*DMD*^+/−^), and male and female wild-type pigs (WT). Tissues of male pigs of different age groups (3 days up to 9 months) were used to build up a muscle bank with myoblasts of DMD pigs and their wild-type siblings.

### 2.2. Isolation of Different Muscle Groups

**Figure 1:**
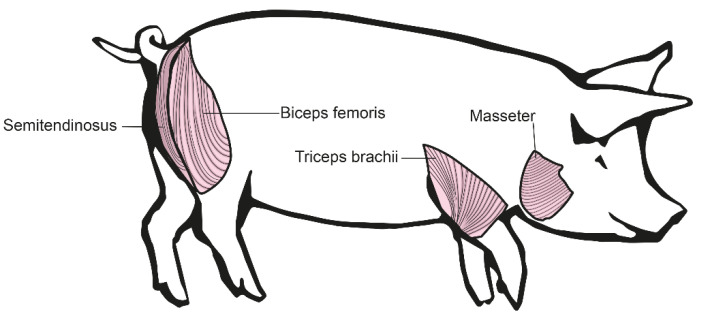
Different muscles used for primary muscle cell culture: *Musculus biceps femoris* and *Musculus semitendinosus* from the pig’s hind leg, *Musculus triceps brachii* from the pig’s front leg, and *Musculus masseter* from the pig’s head.

To adapt for potential differences between muscle groups, we decided to isolate muscle cells from four different muscle groups with different characteristics. These muscles were *Musculus biceps femoris*, *Musculus triceps brachii*, *Musculus semitendinosus*, and *Musculus masseter* (Figure 1).

**Figure 2:**
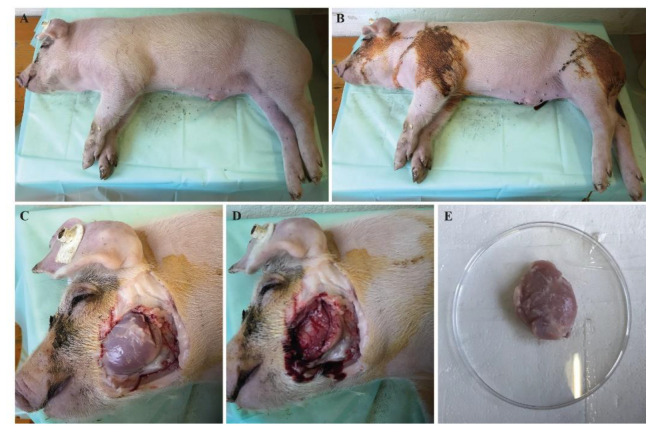
Taking a muscle biopsy of a DMD pig on the example of the *M. masseter*: (**A**) set-up of animal and cleaning with water, (**B**) disinfection of areas of extraction with Braunol^®^, (**C**) skinning of the area, (**D**) area after extraction (left side), (**E**) extracted *M. masseter* in a sterile dish.

Muscle biopsies have been taken within three hours after the death of the respective animal. To ensure sterile conditions, the area of extraction was cleaned with water and then disinfected properly with Braunol^®^ (Figure 2). The extraction of the respective muscle was performed using scalpels. Depending on the age and size of the pig or piglet, the whole muscle or only a part of each muscle were extracted. Each biopsy was divided into two samples, one for cryo-conservation and one to generate the cell culture. Ideally, at least 10 g of each muscle where available. The sample used for cryo-conservation was transferred into a sterile 50-mL centrifugation tube. The other biopsy sample intended for cell culture was also transferred into a 50-mL centrifugation tube completely filled with solution A (30 mM HEPES, 123 mM NaCl, 3 mM KCl, 10 mM glucose, 1 nM phenol red, 100 µg/µL penicillin/streptomycin sulfate, 250 ng/mL amphotericin B).

### 2.3. Isolation of Satellite Cells

The isolation and enrichment of satellite cells were performed according to the adapted protocols from [20,21]. Briefly, the following steps were performed:

- Using a laminar flow cabinet, solution A was removed from the sample.
- Muscle tissue was separated from connective and fat tissue using a sterile scalpel.
- Then, remaining muscle was weighed, a minimum of 3 g, but if possible, 5–20 g of muscle were used for each primary culture.
- The piece of muscle was cut into very small pieces.
- The cut muscle pieces were magnetically stirred for 20 min at 37 °C in a 500 mL Schott^®^ bottle in 25 mL of Hank’s balanced salt solution (HBSS) containing 0.2% collagenase I, 0.01% DNase I, and 0.025% trypsin.
- Following, the bottle was chilled in an ice bath for 2 min.
- The supernatant was transferred into fresh tubes and mixed with an equal amount (1:1) of growth medium (α-MEM, 10% FCS, 1% Glutamax, 0.2% amphotericin B, 0.2% gentamicin). For the remaining muscle piece, the steps from the magnetic stirring to the transfer of the supernatant were repeated two times.
- This mix was filtered through a 100 µm micro strainer and then centrifuged (800× *g*, 10 min, 4 °C).
- The supernatant was discarded and the pellet was resuspended in 15 mL of growth medium (4 °C).
- The suspension was then filtered through a 70 µm micro strainer and centrifuged (800× *g*, 10 min, 4 °C).
- The supernatant was discarded and the pellet resuspended in 12 mL of PBS.
- The suspension was slowly and carefully given on top of a step gradient [(50-mL tube containing 3 mL of a 60% Percoll-solution (Easycoll and PBS, lower layer) and 35 mL of a 20% Percoll-solution (upper layer)].
- This 50 mL tube was centrifuged (4800× *g*, 45 min, 4 °C, no brake)
- The boundary layer between the 20% and the 60% Percoll-solution was taken out by a sterile Pasteur pipette (approx. 8–12 mL), transferred into a new sterile tube, and mixed with 15 mL of growth medium. This suspension was centrifuged (500× *g*, 10 min, 4 °C), the supernatant was discarded, and the pellet resuspended in 10 mL growth medium.
- Depending on the size of the pellet, approx. 1 mL cell-pellet was transferred with 18 mL of additional growth medium into one 75 cm^2^ cell culture bottle.
- The newly harvested satellite cells were grown in growth medium in a 37 °C incubator with 5% CO_2_ for 24 h. The growth medium was changed after one day.
- A mycoplasma infection test was made regularly with the cells, using the MycoAlert^™^ Mycoplasma Detection Kit (LT07-118, Lonza, Basel, Switzerland).

### 2.4. Cell Growth, Storage, and Differentiation

Cells were grown at 37 °C and 5% CO_2_ until they reached a confluency of 60–70%. Passaging cells were split at the confluency 1:2 using trypsin, and during passaging, coverslips were added at this stage for later fixation if necessary. Storage cells were frozen in freezing medium consisting of 90% FCS and 10% DMSO. Cells were brought slowly to −80 °C using Nalgene^®^ Mr. Frosty^TM^ (Thermo Fisher, Waltham, MA, USA) and transferred into liquid nitrogen after 48 h for long-term storage.

For differentiation, dishes were coated using a Matrigel-based coating mixture (0.5% Maxgel ECM solution and 0.04% pig skin gelatine solution in Dulbecco’s modified Eagle’s medium). Myoblasts were seeded in growth medium on the coated plates. At about 80 to 90% confluence, the growth medium was replaced by the differentiation medium (DMEM high glucose with 0.4% Ultroser G, 0.22% amphotericin B, and 0.2% gentamicin). Myotubes were differentiated for 4–12 days, depending on the needs of each project. The differentiation medium was exchanged partially every 2 days.

For the coating experiments, the MicroMatrix™ 36 plate from MicroStem was used. According to the manufacturer’s protocol, the MicroMatrix™ 36 slide was washed once with 5 mL PBS and once with 5 mL growth medium. Then, 150,000 cells were seeded out in 5 mL of growth medium and incubated at 37 °C with 5% CO_2_. After 24 h, the slide (containing 36 different coating areas, each having nine coated spots and including a field as negative control) was imaged. For this experiment, we used myoblasts of a four-month-old DMD pig and its male wild-type sibling at passage 9.

### 2.5. Western Blot

Western blot was performed using BioRad’s Tetra electrophoresis chamber and the TransBlot Turbo^®^ system for the actual protein blotting. Whole cell lysates were generated with RIPA buffer (50 mM Tris/HCl pH 8.0, 150 mM NaCl 1% NP40 0.5% sodium deoxycholate, 0.1% SDS, freshly added protease inhibitor tablet from Roche (Complete Ultra protease inhibitors #05 892 970 001)), a MagNA Lyser (Roche) or a Bandelin Sonopuls ultra sonicator. Then, 25 µg whole protein was used per lane in the different blots. For normalization and quantification, we used GAPDH as a loading control (antibodies Cell Signalling D16H11; Millipore MAB374, 1:10,000). The following primary antibodies were used for the utrophin (Santa Cruz 8A4 #SC-33700, 1:500 in 5% BSA/TBST) or dystrophin (ab15266, 1:500 in 1% CWS/TBST) blots. All Western blots were detected in the LiCor Odyssey Fc Imaging System using fluorescent secondary antibodies (Donkey anti-rabbit 800 CW 926-32213, Donkey anti-mouse 680RD 926-68072, Donkey anti-rabbit 680 RD 926-68073, and Donkey anti-mouse 800 CW 926-32212).

### 2.6. Immunofluorescence Staining

Proliferating cells were fixed on a coverslip at about 70% confluence, differentiating cells at the respective timepoints of interest. The cells were fixed with ice-cold methanol or 4% PFA in PBS for 15 min. Before staining, PFA fixed cells were permeabilized with 0.1% Triton X-100 for 5 min. As a myogenic marker, cells were stained for desmin (NBP1-45143, 1:50; or NSJ-R31530, 1:50). To check the proliferation status, cells were stained for Ki-67 (SAB5500134, 1:1000). To differentiate between myoblasts and fibroblasts, we also stained against fibronectin (SC-59826, 1:50). For myoblast differentiation, we stained at different timepoints for myosin heavy chain (Novocastra^TM^, NCL-MHCf, 1:100) and PCM1 (Sigma HPA023370, 1:100). Immunofluorescence pictures were taken with an Olympus IX83 microscope and U Plan X Apo 40× or U Plan X Apo 10× objective. The cell counter function of Image J [22] was used to determine the number of cells (DAPI), as well as the number of cells which were positive for desmin and/or Ki-67.

## 3. Results

### 3.1. Characterization of Cell Growth and Myogenic Content

**Figure 3:**
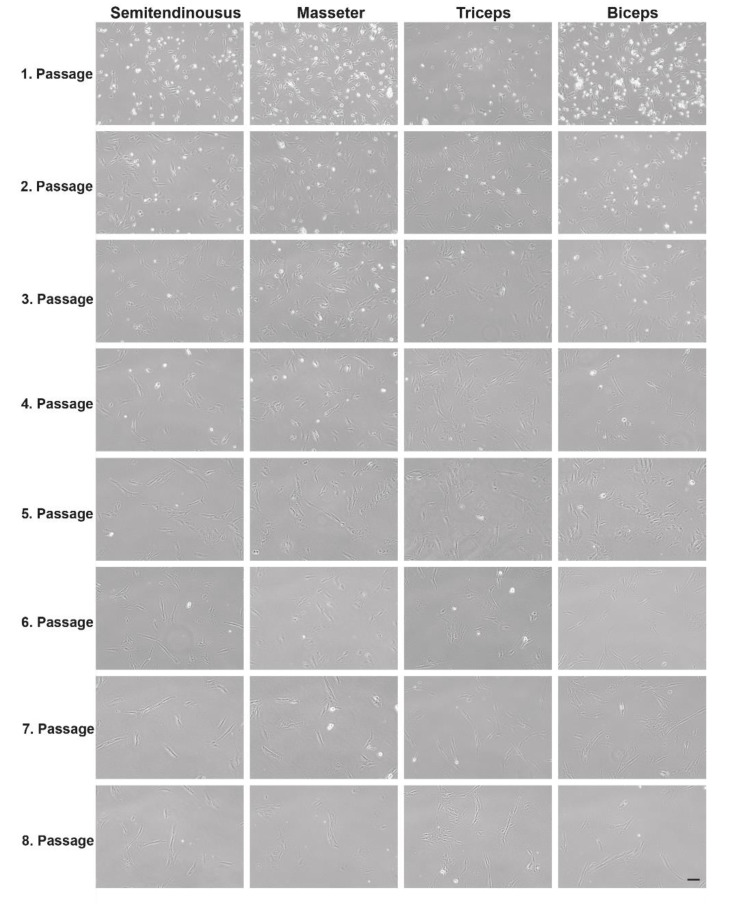
Comparison of cell growth between primary myoblast cultures gained from *Musculus biceps*, *triceps*, *semitendinosus*, and *masseter* of a four-month-old DMD pig. Pictures were taken 24 h after each passaging. Scale bar 100 µm.

One of the main questions was, if there is a difference between the myoblast cultures gained from different muscle types. To investigate this, we monitored the development of the cultures over several passages. We could not see any difference between myoblasts of wild-type pigs and DMD pigs as well as the age of the animals (data not shown). The muscle used as starting material for the myoblast culture had no effect on the development of the culture (Figure 3). Myoblasts of a four-month-old DMD pig and its male wild-type sibling were grown over eight passages. For this, four different muscle types were used from each animal. When the myoblasts reached 60–70% confluence, they were passaged and coverslips were fixed for immunofluorescence staining. Myoblast cultures of all four muscle types grew at the same level and showed no differences in the day-to-day handling. Within all muscle types as well as age groups, the cell size increased in early passages and the speed of growing decreased from passage five onwards (a confluency of 60–70% was reached after 24 h for the first five passages and for passages six–eight, it took 48 to 72 h).

**Figure 4:**
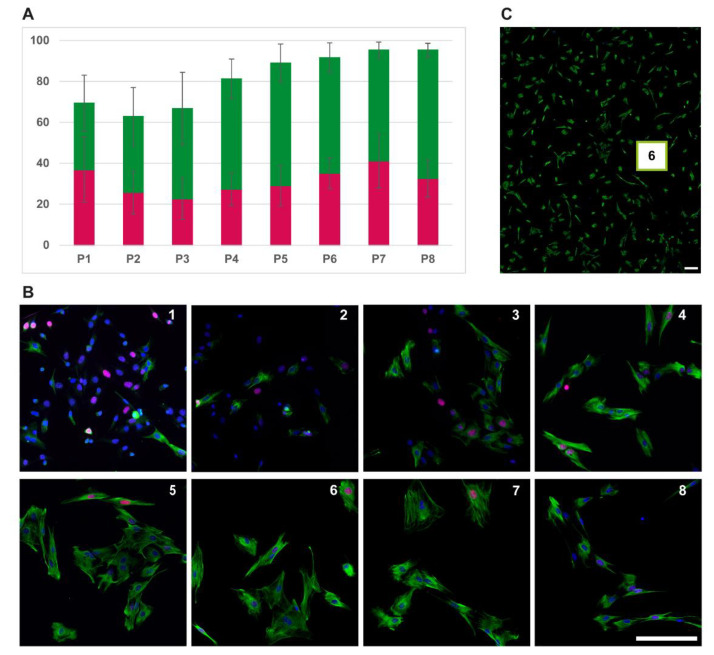
Proliferation over time. (**A**) Percentage of myoblasts (desmin positive cells) over 8 passages (green) and level of proliferating within the myoblasts (positive for Ki-67, red) with standard deviation, (**B**) and cell culture of a DMD pig (*M. biceps femoris*) over 8 passages stained for desmin (green) and Ki-67 (red). Coverslips of each passage were fixed 24 h after each passaging. (**C**) Example for overview pictures (passage 6). Scale bars 200 µm.

To test the myogenic and proliferative character of the cell cultures, we cultivated eight different cell lines (four different muscle types of each, a DMD pig and a wild-type pig) over eight passages. We observed very similar data for all eight cell lines: the percentage of myoblasts increased from roughly 60% up to over 90% in passage seven. The size of myoblasts increased up to passage four and kept then at a constant range. However, from passage seven onwards, the cells grew slower. The number of proliferating myoblasts increased up to passage seven to over 40% and then dropped slowly (Figure 4). There was no difference between the four different muscle types or DMD pigs and wild-type pigs.

### 3.2. Differentiation into Myotubes

**Figure 5:**
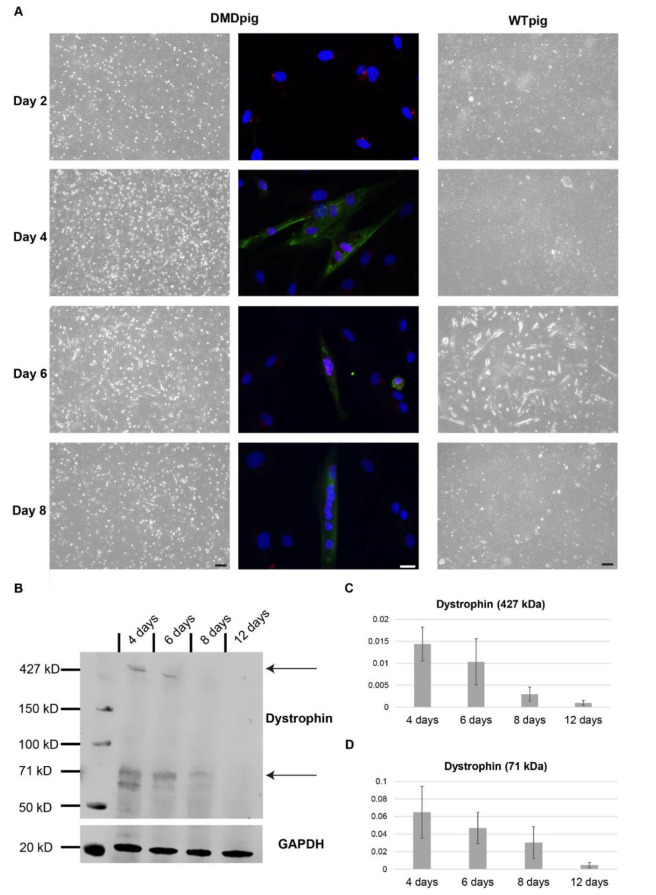
Differentiation of porcine myoblasts and dystrophin expression. The amount of myotubes is the highest between day 3–6 and the dystrophin level decreases with the amount of myotubes from day 6 onwards. (**A**) Pictures taken at day 2, 4, 6, and 8 of differentiation of DMD pig and wild-type pig cells. At the same time points, an immunofluorescence staining was performed with myosin (green) and PCM 1 (red) antibodies to show the level of differentiation in the DMD pig culture. (**B**) Western blot showing the expression of dystrophin in wild-type pig cultures. (**C**) Quantification of full length dystrophin (~427 kDa) and (**D**) a ~71 kDa isoform. Scale bar 200 µm and 20 µm for IF.

One of the most important parts of this project was to develop a DMD cell culture model, which gives measurable outputs to detect the success of possible therapy trials *in vitro*. As the full-length isoform of dystrophin is only starting to get expressed during differentiation, it is necessary to develop a reproducible and reliable protocol for the differentiation of porcine myotubes. To determine the level of differentiation, we performed a co-staining with myosin heavy chain and PCM1. PCM1 (pericentriolar material 1) is a protein of the centrosome that relocates to the outer nuclear envelope during early muscle differentiation [23]. Myosin heavy chains get expressed during muscle differentiation and associate with the cytoskeleton [24]. This allowed the identification of myotubes as well as the number of nuclei within the myotube. We monitored the differentiation for up to twelve days after the start. Up to six days, we could identify myotubes in both, DMD pig and wild-type pig cultures. In wild-type cultures, we observed at 6 days, in general, more and bigger myotubes. (Figure 5A), which we confirmed by fusion index (Supplemental Figure S1). After six days of differentiation, the number of myotubes decreased, which was likely due to detachment of mature myotubes.

The main marker needed in this model is the expression of full-length dystrophin. We performed Western blot on wild-type cultures to identify the level of dystrophin expression during differentiation. We used only wild-type cultures for this as there is no expression of full-length dystrophin in the DMD pig muscle cultures (Supplemental Figure S2). We identified the strongest expression of full-length dystrophin (~427 kDa) as well as a shorter dystrophin isoform (~71 kDa) after four days of differentiation with a decrease of dystrophin afterwards (Figure 5B–D). This correlated with the presence of myotubes.

**Figure 6:**
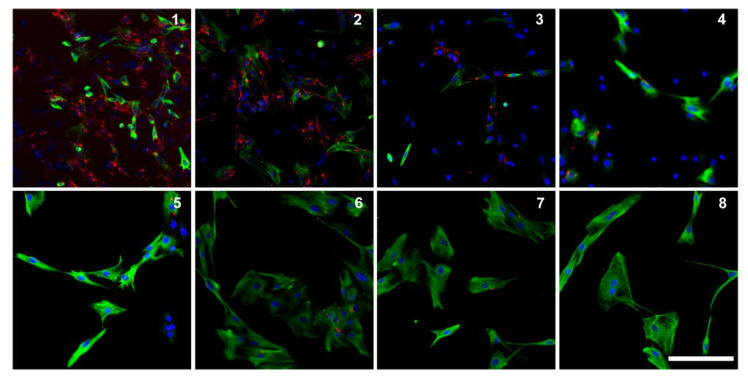
Fibronectin is present in early passages: fibronectin (red) and desmin (green) staining over eight passages of the same cell culture (DMD pig, 4 months old, *M. biceps femoris*). Coverslips have been harvested 24 h after each passaging. Scale bar 200 µm.

We observed differences in differentiation efficiency between the different myoblast cultures, but they were independent of the muscle type used for the biopsy or the age of the animal. The main factor affecting differentiation was the passage number: the lower the passage number, the better the differentiation rate. We got the best differentiation rates during the first four passages. These first passages were also the passages with lower percentage of myoblasts. To investigate if the differentiation efficiency was only due to low passage number or if other factors were involved, we co-stained myoblast cultures for fibronectin and desmin. This showed the presence of fibronectin during early passages, and especially in areas with a strongly positive fibronectin staining, myogenic cells accumulated (Figure 6).

### 3.3. Coating of Dishes

**Figure 7:**
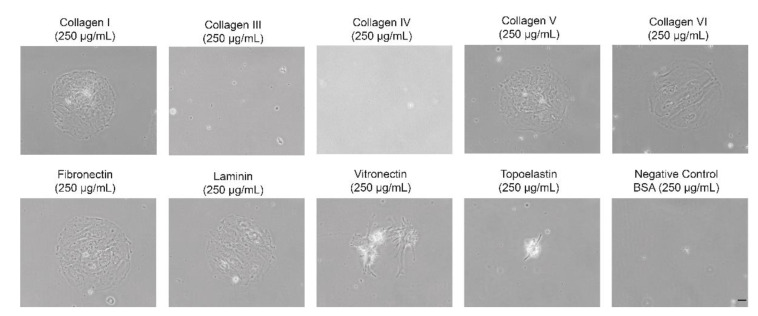
Different coating options confirm fibronectin to be significantly relevant for porcine myoblast proliferation. Nine spots of each coating version were imaged for cell attachment (myoblasts from a four-month-old DMD pig in passage 9 m. biceps femoris) have been imaged 24 h after passaging. Scale bar 20 µm.

To verify the effect of coating on the growth of porcine myoblasts, we tested a set of different coating options. This revealed that the biggest number of cells did attach on fibronectin coating, followed by the collagens type I, V, VI and laminin (Figure 7, complete panel tested in Supplemental Figure S3). Cells attached on fibronectin did have a regular shape. After fixation with PFA and staining with DAPI, counting verified that the highest number of cells attached on fibronectin (Supplemental Figure S4).

### 3.4. Utrophin Expression

**Figure 8:**
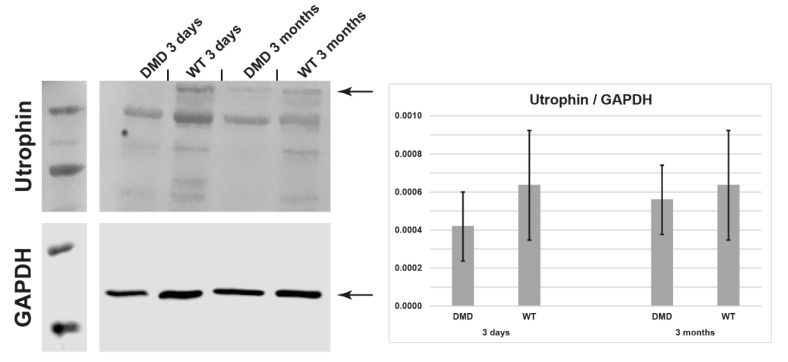
Utrophin expression in myotubes at day 4 of differentiation is not significantly altered in DMD pig myotubes vs. in wild-type myotubes. Myotubes were from a 3-day and a 3-month-old DMD pig (*M. semitendinosus*).

Another important readout is the expression of utrophin. Upregulation of utrophin is thought to be compensatory for the loss of dystrophin in DMD mice, thus explaining the relative mild phenotype of these animals [25,26]. We tested myotubes of 3-day and 3-month-old DMD pigs and their male wild-type siblings for utrophin protein expression by Western blot. In myotubes, utrophin levels do not differ significantly from wild type levels (Figure 8).

## 4. Discussion

The DMD pig represents a superior animal model closely mirroring the human phenotype [19]; therefore, we see the utilization of primary DMD pig muscle cells as an *in vitro* model for preclinical research on DMD as an advancement for the scientific community. The DMD pig muscle cells have the potential to provide a starting point for pipeline therapies from *in vitro* testing in cell culture, *in vivo* testing in an animal model, to application in patients [18]. Usage of DMD pig muscle cells for testing therapies has the benefit that the number of animals for experiments can be reduced by screening for the most promising approaches in a way that is directly transferable to the DMD pig, which has been proven to be an extremely relevant model for DMD [19].

For identification of the optimal muscle for *in vitro* cell culture, we tested four different muscle types with different characteristics. The *musculus biceps femoris* is one of the biggest and strongest muscles of a pig. It is located at the hindquarter of the animal directly under the skin, which makes it easy to extract. Due to its heavy usage, it is likely to show signs of muscle weakness early on. The *musculus triceps brachii* is located at the forehand of the pig. Due to its anatomy, the front legs of a pig carry the most weight and therefore, this muscle needs a lot of training and it is under a lot of pressure. That means the severity of illness should be well detectable here. The *musculus semitendinosus* is a separate muscle located at the hindquarter of the pig, it is easy extractable. The *musculus masseter* is—different to other muscles—served by the cranial nerves and has lots of connective tissue. We found no major differences between the muscle cell cultures gained from these four different muscles. This is of potential benefit, as the amount of starting material is therefore not limited by the size of single muscles. This helps especially with very young animals. We used cells from young animals (3 days old), as we expected fast cell growth in tissue culture. These animals also show a similar phenotype to young DMD boys. Furthermore, we used in this study, mainly cells of animals aged 4 months, as they show a very clear phenotype and thus represent a representative timepoint for the muscle phenotype. Pigs at nine months are fully mature, thus completing the spectrum of ages used.

The disease-relevant protein in DMD is full-length dystrophin, which is only starting to be expressed during muscle differentiation. Therefore, we tested the best differentiation conditions. We got two main results from this: (i) we identified fibronectin to be a major factor in differentiation for porcine myoblasts, which confirms recently published data showing that fibronectin enhances marker expression in porcine myoblasts [27]; and (ii) we demonstrated that the maximum differentiation in porcine muscle cell culture is achieved after only 4 days of differentiation. This is in accordance with the observations of other groups [21]. The positive effect of fibronectin on porcine muscle differentiation shows another potential benefit of this tissue culture model: it is beneficial to use co-cultures, therefore a sorting of cell types is not necessary. The relative fast maximum differentiation time of 4 days might reflect the fast muscle growth in pigs but has the potential benefit to allow a faster workflow in tissue culture.

In summary, we found coating with fibronectin (or fibronectin-containing coating) for tissue culture plates and a shortened differentiation time (compared to other species) provide optimal conditions to investigate full-length dystrophin in primary porcine myoblast cultures. Thus, our *in vitro* DMD pig cell culture model has the potential to speed up results from assays that require differentiation, while there is at the same time more starting material available compared to other species like human (only small muscle biopsies, if any are available) or mouse (in general less muscle mass). Thus, the DMD pig muscle cell culture can improve velocity and cost-effectiveness of animal studies.

## Supplementary Materials

The following supporting information can be downloaded at: https://www.mdpi.com/article/10.3390/life12101668/s1, Figure S1: fusion index of DMD pig and WT pig muscle cultures at 6 days of differentiation.; Figure S2: expression of full-length dystrophin in WT pig and DMD pig myotubes.; Figure S3: complete panel of coating options tested.; Figure S4: quantification of cells attached to coated spots.; Figure S5: full Western blots.
