# Supplementary material for: Isolation and Characterization of Primary DMD Pig Muscle Cells as an In Vitro Model for Preclinical Research on Duchenne Muscular Dystrophy
Source: Life (Basel). 2022 Oct 21;12(10):1668. doi: 10.3390/life12101668 (PMC9604785; doi:10.3390/life12101668)
Supplement: Supplementary file 1 [file life-12-01668-s001.zip › Figure S3.pdf]

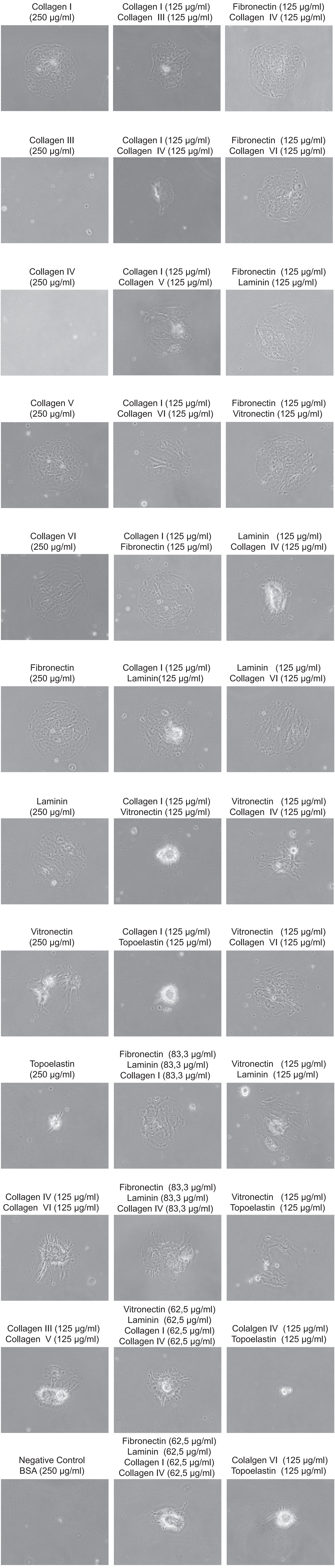
Collagen I (250 µg/ml)
Collagen I (125 µg/ml) Collagen III (125 µg/ml)
Fibronectin (125 µg/ml) Collagen IV (125 µg/ml)
Collagen III (250 µg/ml)
Collagen I (125 µg/ml) Collagen IV (125 µg/ml)
Fibronectin (125 µg/ml) Collagen VI (125 µg/ml)
Collagen IV (250 µg/ml)
Collagen I (125 µg/ml) Collagen V (125 µg/ml)
Fibronectin (125 µg/ml) Laminin (125 µg/ml)
Collagen V (250 µg/ml)
Collagen I (125 µg/ml) Collagen VI (125 µg/ml)
Fibronectin (125 µg/ml) Vitronectin (125 µg/ml)
Collagen VI (250 µg/ml)
Collagen I (125 µg/ml) Fibronectin (125 µg/ml)
Laminin (125 µg/ml) Collagen IV (125 µg/ml)
Fibronectin (250 µg/ml)
Collagen I (125 µg/ml) Laminin(125 µg/ml)
Laminin (125 µg/ml) Collagen VI (125 µg/ml)
Laminin (250 µg/ml)
Collagen I (125 µg/ml) Vitronectin (125 µg/ml)
Vitronectin (125 µg/ml) Collagen IV (125 µg/ml)
Vitronectin (250 µg/ml)
Collagen I (125 µg/ml) Topoelastin (125 µg/ml)
Vitronectin (125 µg/ml) Collagen VI (125 µg/ml)
Topoelastin (250 µg/ml)
Fibronectin (83,3 µg/ml) Laminin (83,3 µg/ml) Collagen I (83,3 µg/ml)
Vitronectin (125 µg/ml) Laminin (125 µg/ml)
Collagen IV (125 µg/ml) Collagen VI (125 µg/ml)
Fibronectin (83,3 µg/ml) Laminin (83,3 µg/ml) Collagen IV (83,3 µg/ml)
Vitronectin (125 µg/ml) Topoelastin (125 µg/ml)
Collagen III (125 µg/ml) Collagen V (125 µg/ml)
Vitronectin (62,5 µg/ml) Laminin (62,5 µg/ml) Collagen I (62,5 µg/ml) Collagen IV (62,5 µg/ml)
Colalgen IV (125 µg/ml) Topoelastin (125 µg/ml)
Negative Control BSA (250 µg/ml)
Fibronectin (62,5 µg/ml) Laminin (62,5 µg/ml) Collagen I (62,5 µg/ml) Collagen IV (62,5 µg/ml)
Colalgen VI (125 µg/ml) Topoelastin (125 µg/ml)
