# Supplementary material for: Isolation and Characterization of Primary DMD Pig Muscle Cells as an In Vitro Model for Preclinical Research on Duchenne Muscular Dystrophy
Source: Life (Basel). 2022 Oct 21;12(10):1668. doi: 10.3390/life12101668 (PMC9604785; doi:10.3390/life12101668)
Supplement: Supplementary file 1 [file life-12-01668-s001.zip › Figure S5.pdf]

Western Blots for Dystrophin

WB # 1

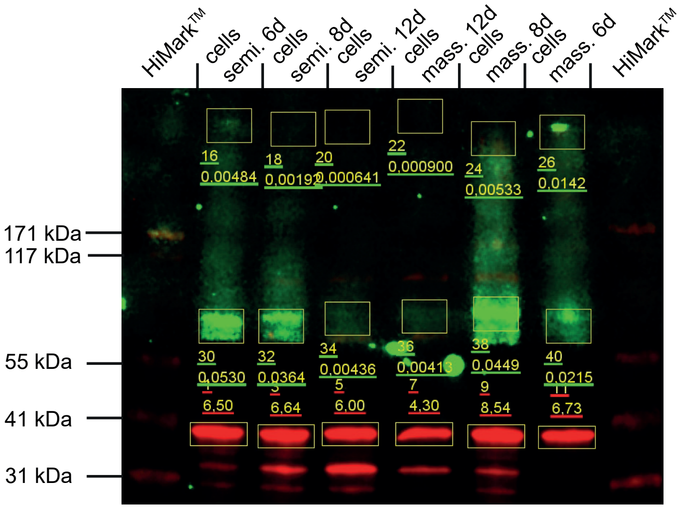

WB # 2

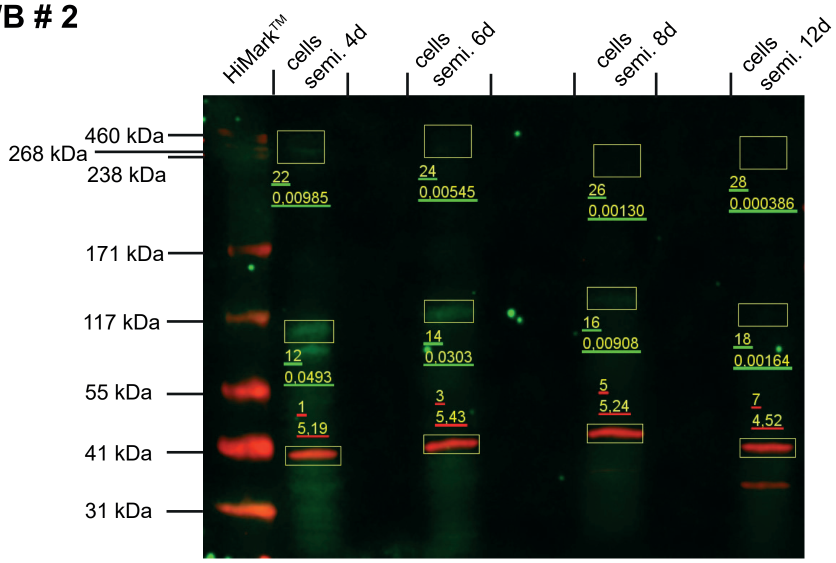

WB # 3

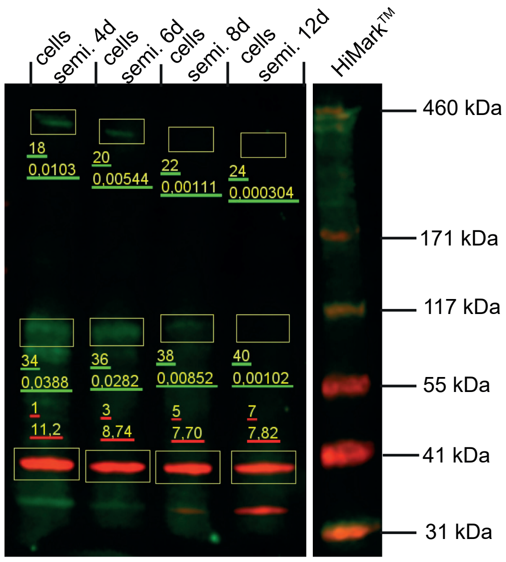

LC5688: HiMark™ Unstained Protein Standard

Western Blots for Utrophin

WB # 4

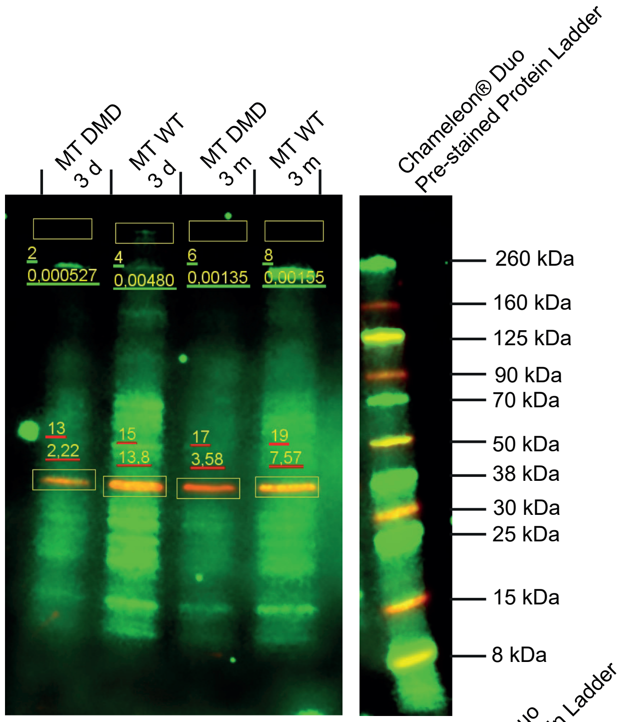

WB # 5

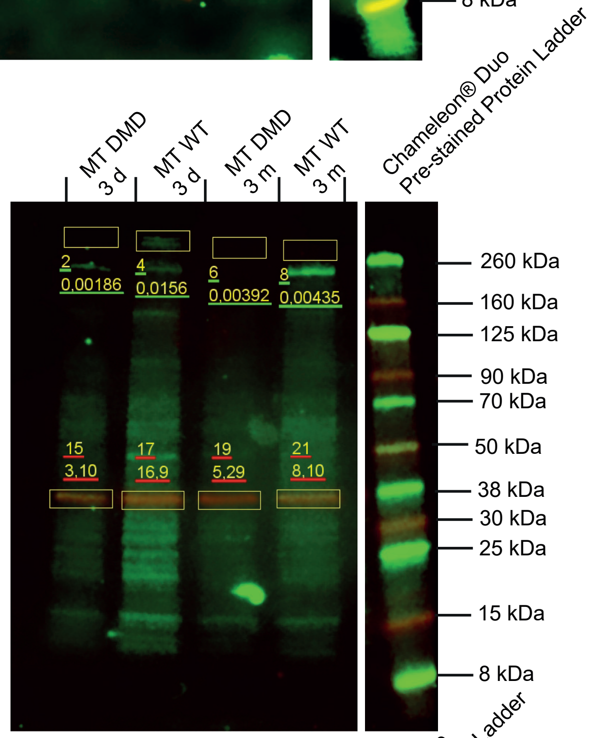

WB # 6

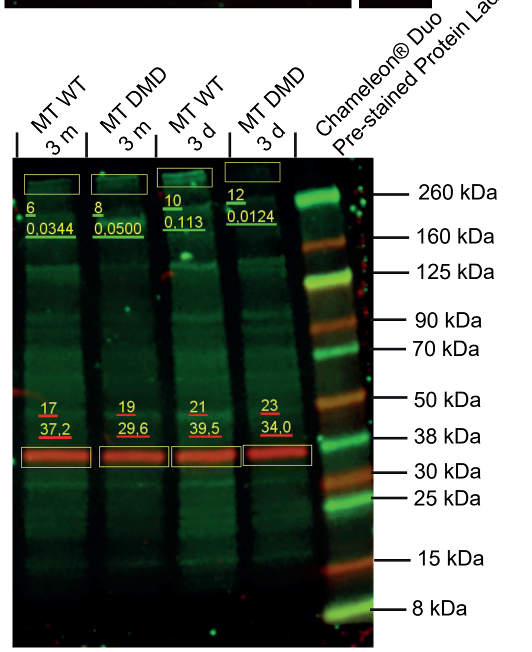

#928 -60000 Chameleon® Duo Pre-stained Protein Ladder
