# Supplementary figures and images for: Isolation and Characterization of Primary DMD Pig Muscle Cells as an In Vitro Model for Preclinical Research on Duchenne Muscular Dystrophy
Source: Life (Basel). 2022 Oct 21;12(10):1668. doi: 10.3390/life12101668 (PMC9604785; doi:10.3390/life12101668)

## Fusion index at day 6

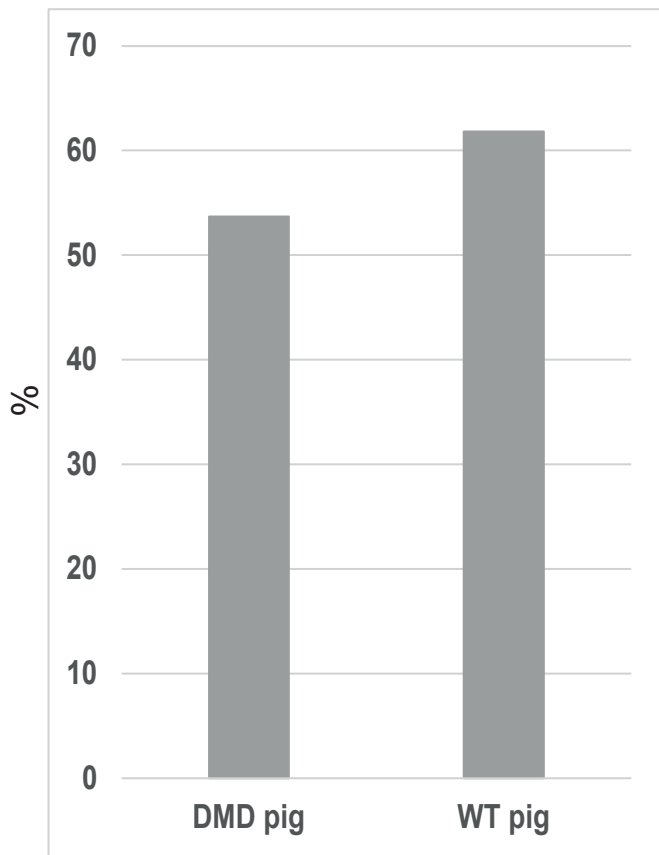

Supplement: Supplementary file 1 [file life-12-01668-s001.zip › Figure S1.pdf]

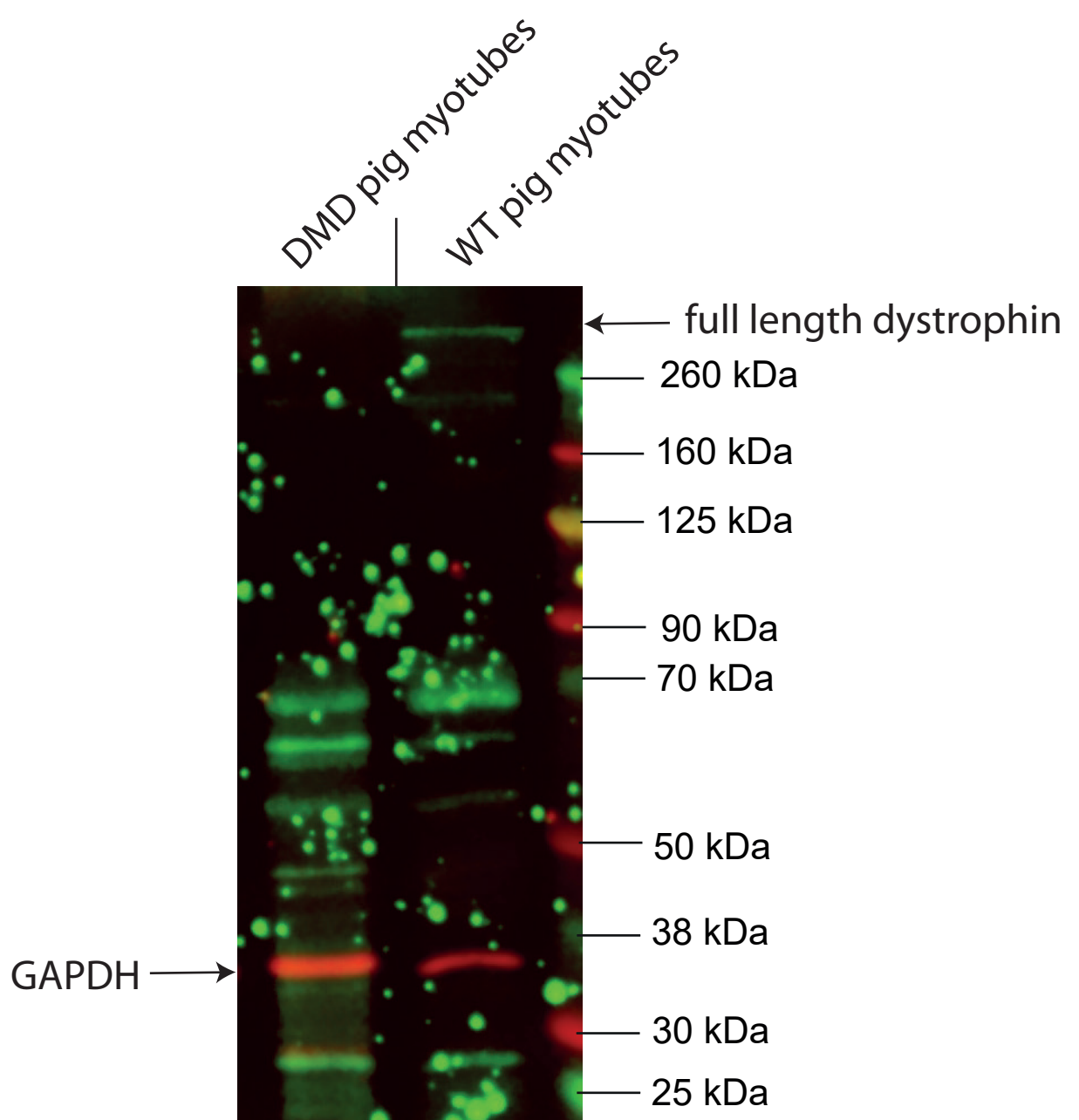

Dystrophin AB with IR800 secondary ab (green)  
GAPDH AB with IR680 secondary ab (red)

Supplement: Supplementary file 1 [file life-12-01668-s001.zip › Figure S2.pdf]

Number of cells per coated spot based on DAPI staining following PFA fixation

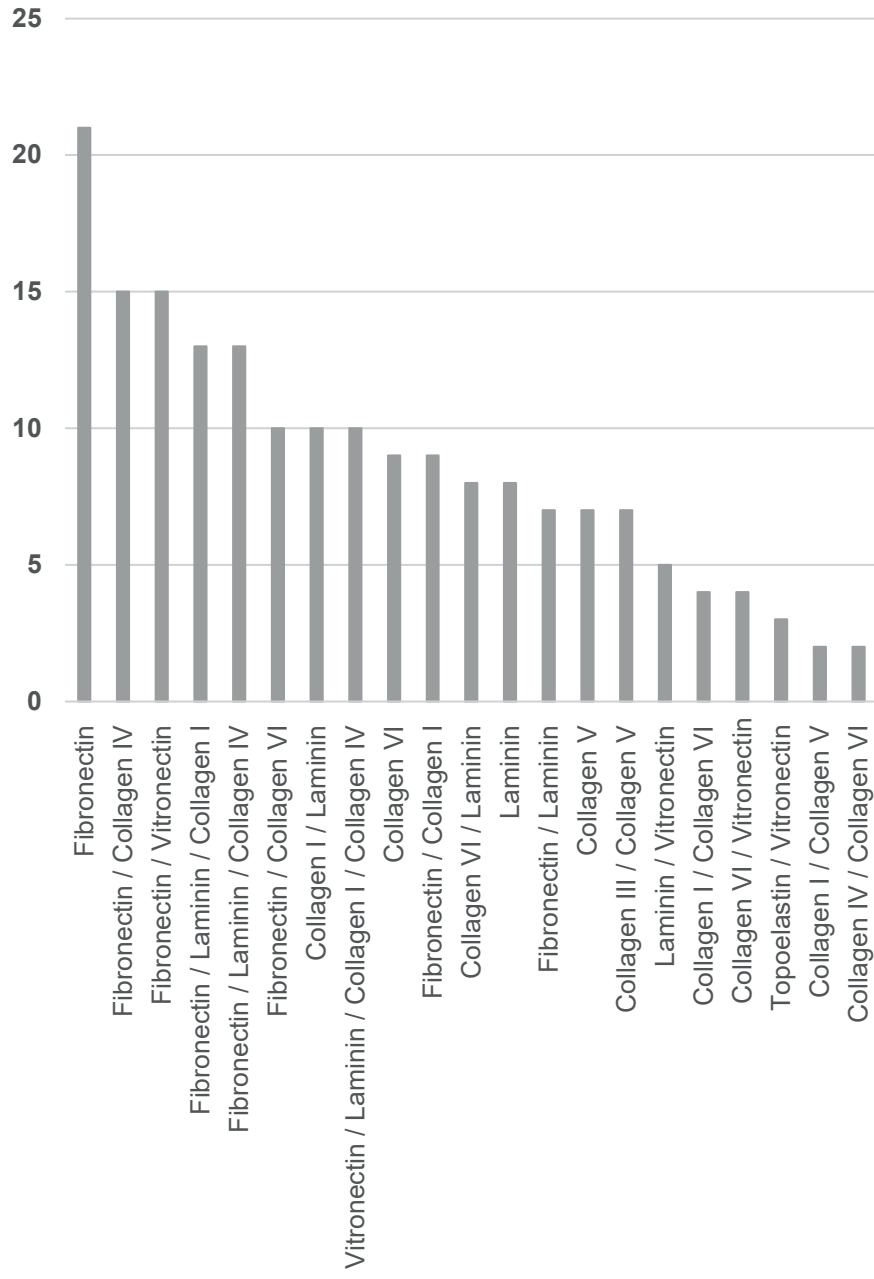

Supplement: Supplementary file 1 [file life-12-01668-s001.zip › Figure S4.pdf]
